# Occult hepatitis B and risk of reactivation following switch to non-hepatitis B virus-active antiretroviral therapy in people with HIV

**DOI:** 10.1097/QAD.0000000000004511

**Published:** 2026-06-25

**Authors:** Giuseppe Lapadula, Alessandro Soria, Massimo Puoti, Paolo Bonfanti

**Affiliations:** a School of Medicine, University of Milano-Bicocca, Milan; b Infectious Diseases Unit, IRCCS Fondazione San Gerardo dei Tintori, Monza; c Infectious Diseases Unit, ASST Grande Ospedale Metropolitano Niguarda, Milan, Italy.

**Keywords:** antiretroviral therapy switch, dual therapy, HBcAb, hepatitis B core antibody, hepatitis B virus reactivation, HIV coinfection, long acting, occult hepatitis B infection

## Abstract

Occult hepatitis B infection (OBI), defined by the persistence of replication-competent hepatitis B virus (HBV) DNA in the liver in the absence of detectable hepatitis B surface antigen (HBsAg), represents a clinically relevant condition among people with HIV (PWH). The long-term persistence of covalently closed circular DNA within hepatocytes provides the biological basis for potential HBV reactivation, particularly after discontinuations of HBV-active antiretrovirals. In recent years, the increasing use of tenofovir-sparing and lamivudine-sparing antiretroviral strategies, including dual and long-acting injectable regimens, has renewed concerns regarding this risk in individuals with serological evidence of prior exposure. We performed a narrative review to summarize current evidence on the epidemiology, biological mechanisms, and clinical relevance of OBI in PWH, with a focus on HBV reactivation following antiretroviral treatment (ART) switches. Evidence from case reports, observational cohorts, and randomized switch trials was examined. Available evidence indicates that HBV reactivation after withdrawal of HBV-active antiretrovirals in HBsAg-negative, antihepatitis B core antibody-positive PWH is a real but infrequent event. Reactivation appears to cluster in specific contexts, including advanced or poorly controlled HIV infection, absence of protective anti-HBs antibodies and a history of HBsAg loss during antiviral treatment. In contrast, in individuals with sustained HIV virological suppression, preserved CD4^+^ cell counts, and markers of effective immune control, the absolute risk of clinically significant reactivation is low. These findings support a risk-based approach to antiretroviral simplification incorporating HBV serological history, vaccination status, and postswitch monitoring, rather than uniform restrictions on treatment switches in PWH with resolved HBV infection.

## Introduction

Hepatitis B virus (HBV) infection remains a substantial clinical and public health burden among people with HIV (PWH), shaping long-term liver outcomes and complicating antiviral management strategies worldwide. This complexity extends beyond chronic HBV to individuals with past HBV infection, in whom possible residual, nonreplicating viral persistence within the liver raises concern that discontinuing antiretroviral regimens containing HBV-active agents may precipitate viral reactivation.

Against this background and in light of the increasing clinical interest in occult hepatitis B infection (OBI) among PWH who use long-acting therapies, we conducted a narrative, nonsystematic review of the available literature.

## Methods

Literature identification was performed through multiple complementary strategies. First, a structured search of PubMed was conducted using relevant Medical Subject Headings (MeSH) related to OBI, hepatitis B virus reactivation, and HIV infection. Second, key review articles addressing OBI in both HIV-infected and non-HIV-infected populations were identified, and their reference lists were manually screened to retrieve additional pertinent studies. Third, we examined inclusion criteria and reported adverse events from randomized controlled trials and observational studies evaluating switches to dual antiretroviral regimens without lamivudine and tenofovir, with particular attention to HBV-related outcomes. Additionally, artificial intelligence-assisted medical knowledge platforms (OpenEvidence, Elicit, Scholar AI) were queried to support identification of relevant literature and contextual evidence.

Titles and abstracts were independently screened by two authors (G.L. and A.S.). Studies were selected for inclusion when concordance was reached regarding their relevance. In cases of disagreement, eligibility was resolved through discussion between the reviewers. The final selection emphasized studies providing insight into OBI prevalence, detection, and clinical consequences in the context of HIV infection and antiretroviral therapy switches.

### Epidemiology of hepatitis B virus infection

In the general population, HBV infection, as evidenced by hepatitis B surface antigen (HBsAg) positivity, shows marked geographic variability, with prevalence rates reaching 10–15% in countries such as those in sub-Saharan Africa, Southeast Asia, and parts of Eastern Europe [1,2]. In contrast, prevalence is less than 1% in the United States and most of Western Europe [1,3]. Among PWH, the prevalence of chronic HBV infection is consistently higher than in the general population, reflecting shared transmission routes (parenteral and sexual). Globally, approximately 8% of PWH are coinfected with HBV, with regional variation: in Africa, coinfection rates exceed 15%, while in North America and Europe, rates are generally lower, around 5–8% [4,5].

From an epidemiological perspective, people with chronic HBV infection (with or without hepatitis), represent the primary reservoir for HBV transmission, as the virus is strictly human and can only be transmitted from individuals with ongoing viral replication. In contrast, the prevalence of antibodies to the hepatitis B core antigen (anti-HBc), reflecting immune recognition of the highly immunogenic core antigen, serves as a marker of previous HBV exposure and is substantially higher. In the US general population between 1999 and 2006, the age-adjusted prevalence of anti-HBc was estimated at 4.7%, corresponding to approximately 11.3 million individuals with evidence of past or current HBV infection [6].

Among PWH, anti-HBc prevalence is markedly higher than in the general population and is influenced by both individual exposure risk factors and the underlying HBV epidemiology of the country. In the United States, anti-HBc positivity has been reported in nearly 50% of PWH [7]. In other countries, reported estimates range between approximately 30 and 70% across different cohorts, largely depending on transmission risk groups and background HBV circulation [8–10]. This large population of individuals with serological evidence of prior HBV exposure, despite apparently resolved infection, constitutes the main substrate from which occult infection and, under specific conditions, HBV reactivation may arise in PWH.

### Replication of hepatitis B virus and persistence in the liver as the biological basis for reactivation

**Fig. 1:**
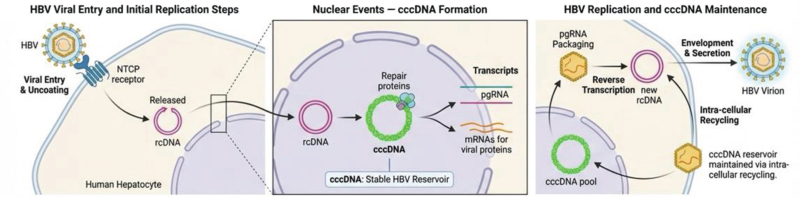
The persistent reservoir of covalently closed circular DNA in hepatitis B virus-infected hepatocytes.

The HBV replicative cycle begins with viral entry into hepatocytes. After uncoating, the relaxed circular DNA (rcDNA) of the virus is transported to the nucleus, where it is repaired and converted into covalently closed circular DNA (cccDNA). This cccDNA forms a stable mini-chromosome that serves as the template for all HBV transcripts, including pregenomic RNA and messenger RNAs for viral proteins [11,12]. As such, cccDNA is central to HBV persistence (Fig. 1). It is highly stable and can persist in hepatocyte nuclei for years or decades, even after apparent clinical resolution of infection or after suppression of viral replication with nucleos(t)ide analogs [13–18]. In addition to cccDNA persistence, HBV replication cycle generates pregenomic RNA, which is packaged into nucleocapsids and reverse-transcribed by HBV polymerase to form new rcDNA. While some nucleocapsids are enveloped and secreted as virions, others recycle rcDNA back to the nucleus to replenish the cccDNA pool, maintaining the reservoir [19].

cccDNA, covalently closed circular DNA; HBV, hepatitis B virus; mRNA, messenger RNA; NTCP, Na (sodium) taurocholate cotransporting polypeptide; pgRNA, pregenomic RNA; rcDNA, relaxed circular DNAirine.

In contrast with CD4^+^ memory T cells in HIV infection, HBV-infected hepatocytes do not survive for the lifetime of the host and are subject to turnover. However, their relatively long lifespan and the stability of cccDNA allow HBV to persist for decades, thereby sustaining the risk of late reactivation. Long-term persistence of infected hepatocytes is also facilitated by the noncytopathic nature of HBV, the tolerogenic liver environment, and the inability of the immune system to completely eliminate all cccDNA-containing cells. In addition, while mitosis of hepatocytes does not efficiently transmit cccDNA to daughter cells, because viral episomes do not seem to have dedicated retention mechanisms, experimental models show that subclinical HBV replication plays a role in maintaining and replenishing the cccDNA pool of quiescent cells by infecting neighboring hepatocytes, even in the absence of detectable HBsAg or HBV DNA in serum [19]. This process can be particularly relevant in the context of immunosuppression or minimal residual viral activity, where new cccDNA molecules can be established without overt clinical or biochemical evidence of infection. Importantly enough, current antiviral therapies have no direct effect on intrahepatic cccDNA, because they act by inhibiting reverse transcription and subsequent steps of viral replication and *de novo* virion formation. Nevertheless, a gradual reduction in cccDNA may occur during long-term therapy as a consequence of hepatocyte turnover and noncytolytic immune control, but eradication of the intrahepatic reservoir is rarely achieved [17,18].

**Fig. 2:**
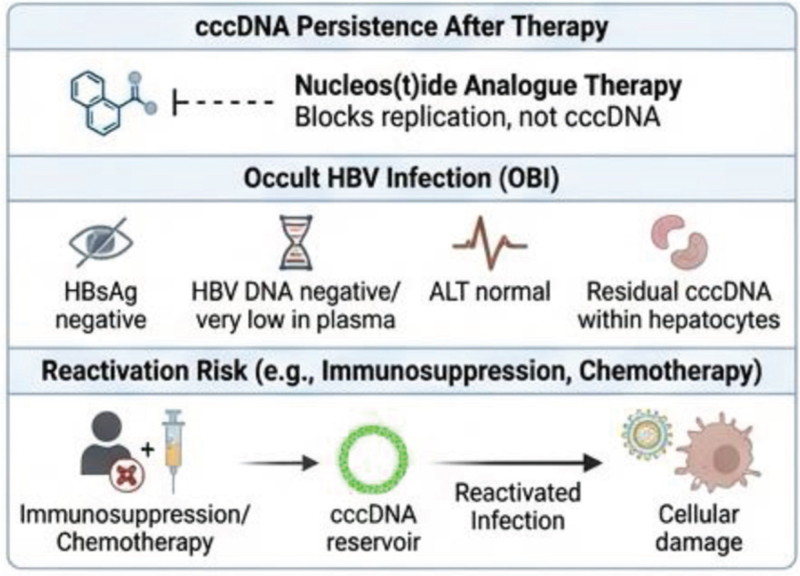
Persistence of hepatitis B virus covalently closed circular DNA as the basis of occult hepatitis B virus infection and reactivation.

ALT, alanine aminotransferase; cccDNA, covalently closed circular DNA; HBsAg, hepatitis B surface antigen; HBV, hepatitis B virus.

Therefore, the persistence of the cccDNA underlies the risk of occult infection – where individuals have undetectable HBsAg, undetectable or very low plasma levels of HBV DNA and normal ALT but retain cccDNA within hepatocytes. In such cases, the infection is not eradicated, and cccDNA remains a reservoir for potential reactivation, especially under immunosuppression or chemotherapy (Fig. 2).

### Occult hepatitis B infection: definitions and diagnostic markers

OBI refers to a condition defined as the presence of replication-competent HBV DNA in the liver, with or without detectable HBVDNA in the blood, in individuals who test negative for HBsAg using currently available assays and, therefore, do not meet the criteria for definition of chronic HBV infection [20]. This residual viral reservoir retains the potential to reactivate under conditions of immunosuppression or immune dysregulation. In this peculiar phase of HBV infection, viral genome persists in the form of cccDNA and in a low state of replication. As a result, the detection of HBV DNA in serum or plasma is intermittent and, when present, typically occurs at very low levels. As a consequence, the reported prevalence of detectable HBV DNA in serum varies widely and is influenced by the characteristics of the population studied, the sensitivity of the assay used, and whether samples are obtained at a single time point or through longitudinal sampling.

Based on the presence of serum markers of prior HBV exposure, OBI can also be classified as seropositive or seronegative [20]. Seropositive OBI, characterized by detectable anti-HBc and/or anti-HBs, is by far the most common form, accounting for more than 80% of cases. In these individuals, HBsAg loss may have occurred after either resolved acute hepatitis B or a long-standing overt chronic infection, and the duration of prior HBsAg positivity before its disappearance may, therefore, be highly variable. Seronegative OBI, much less frequent, is defined by the absence of all HBV serological markers, with HBV DNA – usually detectable only intrahepatically – being the sole indicator of infection.

Despite this conceptual definition, the term OBI has been used in highly variable ways across literature and clinical practice, often leading to confusion and inconsistent classification. In many settings, OBI has been used to describe individuals with detectable HBV DNA in serum despite being HBsAg-negative [21]. Although this scenario does occur, it remains relatively uncommon, compared with the much larger proportion of individuals who harbor HBV DNA within the liver.

A more precise and clinically relevant definition of OBI is the detection of cccDNA in the liver of individuals with negative HBsAg, which identifies those at risk for future HBV reactivation, especially under immunosuppression. Digital droplet PCR (ddPCR) is currently the most sensitive and specific method for the detection and quantification of cccDNA, with a detection threshold that can be lower than 1 copy/μg of extracted DNA [18,22–25]. However, a definition based on HBV-DNA detection in hepatocytes requires liver biopsy, an invasive procedure that is impractical for routine use and subject to sampling constraints.

Given these limitations, a more pragmatic operational definition – sometimes referred to as possible OBI (pOBI) – relies on serological evidence of past HBV infection, most commonly the presence of anti-HBc antibodies. In fact, a substantial proportion of anti-HBc–positive individuals continue to harbor low-level intrahepatic cccDNA despite having no HBsAg or HBV DNA reactivity in serum, placing them within the spectrum of OBI. Across multiple liver tissue-based studies – including analyses of transplant donors, hepatectomy specimens, and pooled meta-analyses – intrahepatic HBV cccDNA has been consistently detected in approximately one half to two thirds of all anti-HBc-positive individuals [22,26–28]. Pooled prevalence estimates across these studies cluster around 50–60%, largely independent of clinical context or the indication for liver tissue availability. In a substantial proportion of anti-HBc-positive individuals, anti-HBs antibodies are also detectable. Observational studies have not demonstrated any correlation between the presence or titer of anti-HBs and the probability of diagnosing OBI among anti-HBc-positive individuals, based on intrahepatic HBV DNA detection [22,28]. However, as discussed in detail later, the absence of anti-HBs reactivity has been associated with an increased risk of HBV reactivation.

In rare instances of past HBV exposure, anti-HBc may be negative despite prior infection, while antibodies directed against other HBV antigens – most commonly anti-HBs – may still be detectable. When anti-HBs is present in an individual whose geographic origin or age group makes prior vaccination unlikely, further evaluation is warranted to determine whether anti-HBs’ positivity reflects prior natural infection rather than vaccine-induced immunity. Indeed, cases of HBV reactivation have been documented in patients with this serological pattern [29].

The definition of OBI has a profound impact on prevalence estimates, and the utility of one definition vs. another depends on the intended purpose and the clinical or epidemiological significance attributed to occult infection. While the detection of HBV genetic material using high-sensitivity nucleic acid testing is entirely reasonable and valuable for identifying hidden infections in the transfusion setting, this approach and definition are inadequate for identifying individuals with a very high risk of reactivation – for example, patients undergoing hematopoietic stem cell transplantation. In such cases, a less specific but more sensitive definition like pOBI, based on serological markers, is required. Table 1 summarizes the different definitions, highlighting their respective advantages and limitations.

**Table 1:** Comparison of definitions used to identify occult hepatitis B infection.

Definition of OBI	Marker/methodology	Pros	Cons	Most useful situations	Estimated prevalence	Ref.
‘Classic’ OBI	HBV DNA+ (in blood or other samples), HBsAg−	Noninvasive	Lower sensitivity than liver tissue; intermittent/low-level viremia may be missed; false negatives possible	Blood donor screening, epidemiological studies, monitoring reactivation risk	<10% of HBcAb+ HBsAg− individuals	[21]
‘Definite’ OBI	cccDNA in liver tissue (ddPCR), HBsAg−	Direct detection of replication-competent virus; highest specificity; identifies true OBI	Invasive; not practical for screening; sampling error; not feasible in most clinical settings	Research, transplant evaluation, high-risk clinical scenarios, definition of HBV ‘cure’	>50% of HBcAb+ HBsAg− individuals	[22,26]
‘Possible’ OBI (pOBI)	HBcAb+, HBsAg−	Widely available; cheap; identifies individuals at risk; useful for epidemiology and risk stratification	Not specific for OBI; some OBI may be HBcAb-negative; cannot confirm active infection	Identifying at-risk populations, guiding prophylaxis in immunosuppression, epidemiological surveillance	HBcAb+ identifies >80% of OBI; 50–66% of HBcAb+ individuals harbor replication-competent HBV DNA in the liver	[20,21,27]

cccDNA, covalently closed circular DNA; ddPCR, digital droplet PCR; HBcAb, hepatitis B core antibody; HBsAg, hepatitis B surface antigen; HBV, hepatitis B virus; OBI, occult hepatitis B infection.

### Prevalence of occult hepatitis B infection among people with HIV

The prevalence of OBI among PWH has been investigated in multiple studies, all of which have adopted the classical operational definition of OBI based on the detection of serum HBV DNA in HBsAg-negative individuals. Overall, the available evidence suggests that HBV DNA positivity is an uncommon but consistently documented condition in this population. However, reported prevalence estimates are highly heterogeneous, reflecting substantial variability in study design, population selection, and laboratory methodologies. Most studies have been retrospective in nature and have relied on convenience samples, enriched for individuals with serological evidence of prior HBV exposure (particularly anti-HBc positivity). In addition, differences in HBV DNA assay sensitivity, thresholds of detection, and the use of single vs. repeated measurements further contribute to the wide range of reported estimates. The calendar period in which cohorts were studied also influences the observed epidemiology of OBI, both because of the increasing proportion of individuals vaccinated against HBV during childhood in more recent cohorts, resulting in lower rates of past HBV exposure, and because of improvements in antiretroviral therapy coverage and effectiveness, which have reduced the frequency of severe immunosuppression and increased the use of regimens containing agents active against HBV.

In earlier studies conducted in the United States, the prevalence of serum HBV DNA positivity evaluated among PWH with pOBI was generally reported to be around 10% [30,31]. More recent cohorts reported lower estimates, with prevalences below 5% [32,33]. Comparable estimates were observed in studies conducted in other high-income countries, such as Italy [34], France [35], and Japan [36]. Conversely, other studies have reported very low or negligible prevalence. In a French cohort of patients with isolated anti-HBc positivity, a prevalence of less than 1% was reported, although approximately two-thirds of participants were receiving antiretroviral regimens with anti-HBV activity [37]. More interestingly, Núñez *et al.* reported no detectable serum HBV DNA among 85 PWH with pOBI who were not receiving tenofovir or lamivudine [38]. These studies highlight how treatment status and study design may substantially influence observed prevalence.

Slightly higher prevalence estimates have been reported in regions with intermediate to high HBV endemicity. Recent data from sub-Saharan Africa support a higher burden of serum HBV-DNA detectability in populations with HIV: reported prevalences include 16% in Ghana [39], 15% in Botswana with much higher detection rates on longitudinal follow-up [40], and 9% in Cameroon in a cohort with a high – though not universal – prevalence of anti-HBc positivity [41].

This heterogeneity in prevalence estimates mirrors that observed in studies conducted in people without HIV, and a prevalence of cryptic HBV DNA (i.e. low-level detectable HBV DNA in HBsAg-negative individuals) of approximately 10% among anti-HBc-positive individuals is in fact comparable to that reported in HIV-negative populations, although direct comparisons are lacking [21].

Among PWH, differences in host immune status and serological profiles appear also to contribute to variability in prevalence estimates. A higher frequency of classical OBI, when defined basing on serum HBV DNA, is expected in individuals with lower CD4^+^ cell counts, suggesting that impaired immune control may facilitate intermittent or low-level viral replication [32,33]. In addition, several studies have observed a higher prevalence of serum HBV DNA detection among individuals with an isolated anti-HBc serological pattern, compared with those who are anti-HBs-positive [32,36,42]. However, not all studies have confirmed a protective role of anti-HBs, and some have found no significant differences in serum HBV DNA-defined OBI prevalence according to anti-HBs status [31]. These associations are biologically plausible in the setting of advanced immunosuppression, where defective HBV-specific cell-mediated immunity likely plays a central role in permitting viral persistence and episodic replication. In contrast, the clinical significance of detectable HBV DNA and the risk of HBV reactivation in individuals who have experienced immune reconstitution under antiretroviral treatment (ART) remain less well defined. This uncertainty is further complicated by the absence of validated biomarkers to assess restoration of HBV-specific cellular immunity: anti-HBs seropositivity, while commonly used as a surrogate, is an imprecise and incomplete marker of effective immune control. Consequently, the risk stratification of HBV reactivation in PWH after immune recovery remains, somewhat, an unresolved issue.

The use of high-sensitivity PCR assays, including ddPCR, has substantially expanded the ability to detect extremely low levels of circulating HBV DNA also among PWH. In a cohort of PWH with pOBI, cryptic HBV DNA was detected in almost 30% of participants using ddPCR, despite the fact that all of them were receiving ART including more than one anti-HBV drug [43]. Notably, comparable findings have been reported in HIV-negative populations: in individuals with pOBI but without HIV, using highly sensitive molecular techniques, HBV DNA was detectable in approximately 40% of cases [44]. It should be emphasized, however, that clinical correlation studies suggest that this form of ultra-low-level viremia is generally not associated with overt liver disease and that the risk of HBV reactivation among individuals with HBV DNA detectable only by highly sensitive PCR is much likely to be comparable to that of individuals in whom HBV DNA is confined to the liver.

Nevertheless, the demonstration of persistent replication-competent HBV in a substantial proportion of HBsAg-negative individuals may explain the growing concerns regarding HBV reactivation when HBV-active agents – most notably tenofovir – are withdrawn, an issue of growing relevance in the current era of increasingly widespread and patient-attractive tenofovir-sparing ART regimens.

### Hepatitis B virus reactivation following withdrawal of hepatitis B virus-active antiretrovirals

As with OBI, definitions of HBV reactivation vary across studies, complicating comparisons and risk estimation. In individuals with pOBI, HBsAg seroreversion is universally considered evidence of reactivation. However, definitions based on HBV DNA detection are more heterogeneous and may influence outcome ascertainment. The American association for the study of liver diseases defines reactivation as either HBsAg seroreversion or newly detectable HBV DNA, without specifying a threshold [45], whereas European association for the study of the liver proposes a more conservative definition including a minimum HBV DNA level of 100 IU/ml [46]. Although such definitions facilitate standardized reporting, the clinical significance of events defined solely by low-level or transient HBV DNA detectability remains uncertain. In this review, the definition of reactivation follows that adopted in each study; wherever possible, we distinguish events defined by low-level HBV DNA detection from those clearly corresponding to HBsAg seroreversion.

Regardless of the definition, the theoretical risk of HBV reactivation after discontinuation of anti-HBV agents is generally considered low in individuals with resolved HBV infection and in the absence of additional risk factors for reactivation. In particular, the risk appears limited in patients without severe immunosuppression, whereas it becomes clinically relevant in settings characterized by profound immune dysfunction, such as advanced HIV disease, hematopoietic stem cell transplantation, or the use of immunosuppressive drugs. However, in the setting of PWH, several case reports and case series have been published, with renewed interest emerging in parallel with changes in ART strategies and the increasing use of nucleoside-sparing regimens, including dual therapies and long-acting injectable combinations. Table 2 summarizes the characteristics of these reactivation cases following ART switches, including patient profiles and risk factors, as reported in published case reports.

**Table 2:** Published case reports of hepatitis B virus reactivation in people with HIV after antiretroviral treatment modification.

Study: first author (year)	CD4^+^ cell count	Risk factors noted	Serology at baseline	ART before and after switch	Time to reactivation	Clinical manifestation	HBVDNA peak (method)	Outcome
Altfeld (1998) [47]	120	Advanced HIV, detectable HIVRNA	HBsAg−, HBsAb+	Zidovudine, **lamivudine**, indinavir to stavudine, didanosine, indinavir	3 weeks	HBsAg seroreversion, transaminase flair	10^9^ copies/ml (nested PCR)	Resolved (serologic resolution) following lamivudine reintroduction
Chamorro (2005) [48]	542	Detectable HIVRNA, nevirapine introduction	HBsAg− HBcAb+ HBsAb−	Zidovudine, **lamivudine** to stavudine, nevirapine, nelfinavir	4 months	Symptomatic hepatitis, HBsAg seroreversion, transaminase flair	>1.7 × 10^7^ copies/ml (hybridization assay)	Resolved following switch to tenofovir + lamivudine
Palmore (2009) [49]	770	Virological failure	HBsAg− HBcAb+ HBsAb−	Lamivudine-containing ART to abacavir, efavirenz, amprenavir, ritonavir	3 months	Symptomatic hepatitis, HBsAg and HBeAg seroreversion, transaminase flair	2.2 × 10^8^ copies/ml	Improvement after lamivudine reintroduction, HBVDNA undetectable, lost after 2 years
Bagaglio (2010) [50]	233	ART discontinuation, CD4^+^ cell drop, detectable HIVRNA	HBsAg− HBcAb+ HBsAb− HBVDNA 1400 copies/ml	Abacavir, **lamivudine**, amprenavir, ritonavir (therapy discontinuation)	3 months	HBsAg seroreversion, transaminase flair	62,000 copies/ml	Transaminase reduction and HBVDNA reduction following switch to tenofovir (patient lost to follow-up)
Bloquel (2010) [51]	220	CD4^+^ cell drop, recent virological failure	HBsAg− HBcAb+ HBsAb−	**Tenofovir, emtricitabine**, lopinavir, ritonavir, enfuvirtide to raltegravir, darunavir, ritonavir, etravirine	6 months	Symptomatic hepatitis, HBsAg seroreversion, transaminase flair	10^8^ copies/ml (RT-PCR)	Resolved following switch to tenofovir + lamivudine
	433	Virological failure	HBsAg− HBcAb+ HBsAb+	Abacavir, **lamivudine**, fosamprenavir, ritonavir, enfuvirtide to raltegravir, darunavir, ritonavir, etravirine	1 month	Transient HBVDNA rise, normal transaminase	10^4^ copies/ml (RT-PCR)	HBVDNA undetectable spontaneously (no ART change)
Costantini (2011) [52]	291	Low adherence, ART discontinuation, CD4^+^ cell drop	HBsAg− HBcAb+ HBsAb−	Atazanavir, **lamivudine** (therapy discontinuation)	4 months	Symptomatic hepatitis, HBsAg seroreversion, transaminase flair	88,185 copies/ml	Resolved following switch to tenofovir + lamivudine
Seang (2013) [55]	>500	None	HBsAg− HBcAb+ HBsAb−	**Tenofovir**, **emtricitabine**, darunavir, ritonavir to raltegravir, darunavir, ritonavir	1 month (HBVDNA), 5 months (HBsAg, hepatitis)	HBsAg and HBeAg seroreversion, transamianse flair	10^8^ copies/ml (RT-PCR)	Resolved following reintroduction of tenofovir and emtricitabine
Mican (2021) [53]	110	Advanced HIV	HBsAg− HBcAb+ HBsAb−	**Tenofovir**, **emtricitabine**, darunavir, ritonavir to **emtricitabine**, darunavir, ritonavir	3 months	Symptomatic hepatitis, HBsAg and HBeAg seroreversion, transaminase flair	4 × 10^6^ copies/ml	Resolved following reintroduction of tenofovir
Vasishta (2023) [54]	13	Low adherence, ART discontinuation, advanced HIV	HBsAg− HBcAb+ HBsAb+	**Tenofovir**, **emtricitabine**, atazanabir, ritonavir to dolutegravir, darunavir, cobicistat	5 months	HBsAg seroreversion, transaminase flair	1.4 × 10^8^ copies/ml	Unknown
Adachi (2024) [56]	512	None	HBsAg− HBcAb+ HBsAb+−	**Tenofovir**, **emtricitabine**, lopinavir/ritonavir to cabotegravir, rilpivirine	4 months	HBsAg seroreversion, transaminase flair	1.5 × 10^8^ copies/ml	Unknown
Olcott (2025) [57]	530	Previous HBV reactivation	HBsAg− HBcAb+ HBsAb−	**Tenofovir**, **emtricitabine**, efavirenz to dolutegravir, rilpivirine	4 months	HBsAg and HBeAg seroreversion, transaminase flare	616,594,900 IU/ml	Unknown
	510	Ageing-associated immunosenescence	HBsAg− HBcAb+ HBsAb+	**Tenofovir**, **emtricitabine**, raltegravir to doravirine, dolutegravir	12 months	HBsAg and HBeAg seroreversion	616,594,900 IU/ml	Unknown
	Unknown (nadir 300)	None	HBsAg− HBcAb+ HBsAb+	**Tenofovir**, **emtricitabine**, doravirine to cabotegravir, rilpivirine	11 months	HBsAg and HBeAg seroreversion, transaminase flare, jaundice	83,176 IU/ml	ALT improved on starting tenofovir
	1100	Splenectomy	HBsAg− HBcAb+ HBsAb−	**Tenofovir**, **emtricitabine**, darunavir/ritonavir to lamivudine, dolutegravir	10 months	HBsAg and HBeAg seroreversion	30,903 IU/ml	Unknown

ART, antiretroviral treatment; HBcAb, hepatitis B core antibody; HBsAb, hepatitis B surface antibody; HBsAg, hepatitis B surface antigen; HBV, hepatitis B virus; RT-PCR, real time PCR. HBV active agents are highlighted in bold.

The first reported cases of HBV reactivation following lamivudine discontinuation in PWH date back to the 1990s. The initial case described by Altfeld *et al.* [47] occurred in a patient with a CD4^+^ cell count below 200 cells/μl. Subsequent reports involved patients with higher CD4^+^ cell counts, but in the context of HIV virological failure due to suboptimal or interrupted antiretroviral therapy, often accompanied by CD4^+^ T-cell declines [48–52]. Analogous cases of HBV rebound in the context of significant immunosuppression have also been reported following tenofovir discontinuation [53,54]. These reported cases share a rapid onset with HBsAg seroreversion occurring within the first few months after discontinuation, and an equally swift response to reinitiation of anti-HBV therapy, with HBsAg returning to negative and no progression to chronic infection, at least in cases where follow-up data are available. These observations reinforce the concept that immune competence plays a critical role in controlling OBI, in the absence of anti HBV treatment.

Conversely, Seang *et al.* reported a seroreversion of HBsAg following discontinuation of tenofovir disoproxil fumarate/emtricitabine in a patient with prior resolved HBV infection who had positive HBsAb and, unlike previous cases, no other concomitant risk factors. This case, while sharing a clinical course consistent with earlier reports, demonstrates that OBI reactivation may also occur in patients with protective anti-HBs titers. Notably, HBV DNA reappeared rapidly – within 1 month of discontinuation – and preceded HBsAg seroreversion and clinical hepatitis by several months, suggesting that close postswitch monitoring may allow for early identification of HBV reactivation [55]. Similarly, a recent case of HBV reactivation in a patient with pOBI, borderline HBsAb titer and a high CD4^+^ cell count was reported following a switch to long-acting cabotegravir/rilpivirine [56]. The patient had previously achieved apparent HBV recovery under continuous tenofovir/emtricitabine, which was never discontinued prior to the switch. The presence of E164V vaccine-escape mutation may indicate that, in this case, HBV had evolved into a replication-competent immune-escape variant, reemerging following withdrawal of anti-HBV therapy. Taken together, these findings suggest that apparent HBV resolution may be, in some instances, therapy-dependent rather than the result of durable immune control. This is further illustrated by a recent case series of HBV reactivations after discontinuation of tenofovir-containing ART, in which viral sequencing in some cases revealed HBsAg escape mutations [57]. The presence of these variants, associated with immune and diagnostic escape, suggests that at least some events may reflect previously unrecognized ongoing infections rather than true HBV reactivation after treatment switch.

Transient episodes of low-level HBV DNA detectability have also been reported after switching from tenofovir-containing ART to long-acting cabotegravir plus rilpivirine when systematic HBV DNA testing was performed. In these reports, individuals did not develop HBsAg seroreversion, and HBV DNA spontaneously became undetectable even without reintroduction of HBV-active therapy [58]. Such findings are difficult to classify as true HBV reactivation and raise questions about the clinical significance of very low-level, transient viremia, as well as the appropriateness of assigning clinical relevance to these events given their benign course and spontaneous resolution. In this regard, cohort data reported by Salpini *et al.* further question the clinical relevance of low-level HBV DNA detectability after tenofovir withdrawal [59]. In a cohort of 101 people with HIV and pOBI, circulating HBV DNA and HBV RNA were assessed using highly sensitive ddPCR. While on tenofovir-containing ART, more than half of participants already had detectable plasma HBV RNA or HBV DNA, although below the limit of standard commercial assays. Twelve months after discontinuation of tenofovir, with most individuals (72%) remaining on lamivudine-containing regimens, 12.9% of the individuals had an HBV DNA above 10 IU/ml. However, HBV DNA levels consistently remained very low, in the range of 10^2^ copies/ml, and no cases of HBsAg seroreversion were observed. Persistent low-level HBV DNA positivity was independently associated with reduced CD4^+^ T-cell recovery. These findings, while suggestive of ongoing HBV replicative activity, indicate that the detection of very low-level viremia – particularly when assessed using ultrasensitive methodologies – should be interpreted with caution, as its clinical significance remains uncertain.

While case reports clearly indicate that HBV reactivation may occur after discontinuation of HBV-active antiretroviral therapy, they do not permit reliable estimates of its incidence, particularly under controlled conditions such as sustained HIV virological suppression and preserved CD4^+^ cell counts. More informative data on the frequency of HBV reactivation following treatment switches in routine clinical practice are provided by case series and cohort studies, which are summarized in Table 3.

**Table 3:** Hepatitis B virus reactivation after discontinuation of hepatitis B virus-active antiretroviral therapy in people with HIV: data from case series and cohorts.

Study: first author (year)	Type of study	Population	Number/rate of reactivation	Serology at baseline	CD4^+^ cell count at baseline	Antiretroviral treatment	Definition of reactivation	HBVDNA peak (method)
Bloquel (2010) [51]	Cross sectional	41	2 events	HBsAg- HBcAb+ HBsAb+/−		No drugs with anti-HBV activity	HBVDNA detectable	10^3^ copies/ml (RT-PCR)
Rouphael (2022) [60]	Aggregated series	696	0.019/100 PYFU	HBsAg− HBcAb+ HBsAb+/−	280 (198–438)	3 no ART 2 stopped 3TC 1 stopped TDF	HBVDNA detectable (2 transaminase flair)	10^9^ copies/ml
Abdullahi (2018) [62]	Cohort	60	6 events 109/1000 PYFU	HBsAg− HBcAb+ HBsAb+/−	508 (274–637)	TDF + 3TC + bPI to bPI monotherapy (darunavir/ritonavir)	HBVDNA detectable (no increase of transaminase)	10^2^ IU/ml
Denyer (unpublished) [66]	Cohort	5954	39 0.7%	HBsAg− HBcAb+ HBsAb+/−	15% <200 38% 200–499 40% ≥500 8% unknown	Continuing ART without anti-HBV activity	Any new HBsAg+ and/or newly detectable HBVDNA 12/37 (32%) ALT increase >100 16/39 (41%) hospitalized	Not available
Salpini (2025) [59]	Cohort	101	13 12.9%	HBsAg− HBcAb+ HBsAb+/−	648 (463–809)	TDF or TAF stop (72.3% switched to a 3TC-including ART)	HBVDNA >10 copies/ml	<10^2^ IU/ml median (IQR) 31 (15–73)
Dieterich (2025) [61]	Cohort	1071	Incidence rate: 0.18 (0.08–0.40)	HBcAb+ HBsAb−		TFV interruption 48% without HBV-active drug	Reactivation (HBsAg seroreversion or HBVDNA+) Hepatitis flare was rare (<1%)	Not reported
		3808	incidence rate: 0.01 (0.00–0.06)	HBcAb+/HBsAb+		TFV interruption 50% without HBV-active drug	Reactivation (HBsAg seroreversion or HBVDNA+) Hepatitis flare was rare (<1%)	Not reported
Gagliardini (2025) [63]	Cohort	91	None reported	HBcAb+	Not reported	Switch to CAB/RPV	Reasons for discontinuation (HBV not systematically assessed)	Not applicable
Mezzadri (2025) [64]	Cohort	40	0	Hába+ HBsAb+/−	721 (357–1055)	Switch to regimens without HBV-drugs	HBVDNA+ (triggered by transaminase elevations)	Not applicable
		59	0	HBcAb+ HBsAb +/−	764 (429–938)	TFV discontinuation, switch to regimen with 3TC/XTC	HBVDNA+ (triggered by transaminase elevations)	Not applicable
Foncillas (2026) [65]	Cohort	184	0	HBsAg− HBcAb+ (90% HBsAb+)	722 (568–933)	Switching to CAB/RPV	HBsAg+ or HBVDNA+ (testing triggered by transaminase elevations)	Not applicable

3TC, lamivudine; ART, antiretroviral treatment; BIC, bictegravir; CAB, cabotegravir; RPV, rilpivirine; TAF, tenofovir alafenamide; TDF, tenofovir disoproxil fumarate.

Several studies have assessed the risk of HBV reactivation after withdrawal of HBV-active agents in cohorts of patients with pOBI. In one of the largest aggregated series, 24 cases of HBV reactivation were described (six new cases among 696 patients with pOBI from the HIV Atlanta Veterans’ Affairs Cohort Study and 18 from literature case reports), estimating an incidence rate of 0.019 cases per 100 person-years [60]. Most of the cases, however, occurred in individuals not receiving ART and only a few occurred after discontinuation of lamivudine (two) or tenofovir (one), while one case occurred in the presence of a probable emerging lamivudine resistance. Similarly, a more recent US cohort, based on electronic health records spanning 2001–2023, estimated very-low overall reactivation rates after tenofovir discontinuation, but these were markedly higher in patients with isolated HBcAb than in those with pOBI and anti-HBsAb positivity (0.67 vs. 0.08 per 100 person-years) [61]. However, the study used a composite definition of reactivation (HBsAg seroreversion or detectable HBV DNA at any level); when restricting the outcome to clinically more relevant events (HBsAg seroreversion or HBV DNA positivity accompanied by hepatitis flare), the incidence decreased to 0.18 vs. 0.01 per 100 person-years.

In contrast, a study from Cameroon reported a markedly higher risk, with 6 of 60 (10%) participants with pOBI showing very-low level, often transient HBV DNA in stored serum samples, corresponding to 109 cases per 1000 person-years [62]. Again, because reactivation was defined solely by any detectable HBV DNA without HBsAg seroreversion or a clinically meaningful threshold, this study likely overestimates the true risk and highlights the limitations of using viremia alone to define reactivation.

Recent observational cohort studies have evaluated outcomes following switches to antiretroviral regimens lacking HBV-active agents, predominantly cabotegravir/rilpivirine. Across more than 300 individuals with pOBI, no HBV reactivation was observed [63–65]. In these studies, HBV testing was performed only in the presence of clinical or biochemical signs of hepatitis flare, which may have reduced sensitivity but rendered the findings more directly relevant to clinical practice.

Finally, a large US Veterans cohort reported higher rates of HBV reactivation after switching to ART without HBV-active drugs (115/7081; 1.6%). However, these data are currently available only as a conference abstract and have not undergone peer review [66]. Given their prominence in the current debate, we cite these findings while acknowledging the uncertainties and inconsistencies in the available report.

Another way to estimate both the incidence and the clinical relevance of HBV reactivation after discontinuation of HBV-active ART is provided by randomized controlled switch trials in which at least one study arm omitted agents with anti-HBV activity. As summarized in Table 4, across a large number of trials conducted over more than a decade, HBV reactivation or acute hepatitis B events were uncommon and generally rare, despite the inclusion of substantial proportions of anti-HBc-positive participants in several studies. Importantly, many trials applied additional restrictions based on anti-HBc, anti-HBs, and HBV DNA status, thereby selecting populations at relatively low baseline risk. Within this context, anyway, cases of acute hepatitis B or virologically documented HBV reactivation were sporadically reported, often as isolated events, and were more frequently observed in study that included also individuals without protective HBsAb antibodies. Nevertheless, the absolute number of events remained very small relative to the total number of participants exposed, and severe liver toxicity was infrequent and usually comparable between experimental and control arms.

**Table 4:** Reactivation/acute hepatitis B virus infections in randomized controlled trial comparing regimens with or without hepatitis B virus activity.

Trial name (year)	HBV exclusion criteria	Treatment arms	*N* per arm	Grade ≥3 liver function test elevation	Acute B hepattis cases
EARNEST (2014) [70]	Known HBsAg+ (testing not required at screening)	Switch to LPV/r+RAL versus PI/r monotherapy vs. PI/r + NRTIs from failing 1st line ART	433 (RAL arm), 418 (monotherapyarm), 426 (NRTI arm)	1 in RAL arm vs. 3 in monotherapy arm vs. 3 in NRTI arm (including acute hepatitis)	3 hepatitis B in RAL arm vs. 2 in monotherapy arm vs. 0 in NRTI arm
NEAT001 (2014) [71]	Excluded if HBsAg+	DRV/r+ RAL vs. DRV/r + TDF/FTC (ART naïve)	401 (RAL), 404 (TDF/FTC)	12 (3%) in RAL arm vs. 4 (1%) in TDF/FTC arm	1 hepatitis B in RAL arm vs. 0 in TDF/FTC arm
LATTE (2015) [72]	Excluded if HBsAg+ or HBcAb+ HBsAb− HBVDNA+	Switch to oral CAB (various dosing)+RPV vs. continue EFV+2 NRTI	181 (CAB+RPV), 62 (EFV)	3 (2%) in CAB arms vs. 1 (2%) in EFV arm	0 in all arms
LATTE-2 (2017) [73]	Excluded if HBsAg+ or HBcAb+ HBsAb− HBVDNA+	Switch to long acting CAB+RPV (various frequency) vs. continue DTG+ABC/3TC	230 (long acting), 56 (oral ART)	8 (3%) in long acting arms vs. 3 (5%) in oral arm	0 in all arms
SWORD-1 and SWORD-2 (2018) [74]	Excluded if HBsAg+ or HBcAb+ HBsAb−	Switch to DTG + RPsV vs. continue current ART	516 (DTG+RPV), 512 (current ART)	2 (<1%) in DTG+RPV arm vs. 1 (<1%) in current ART arm	None reported
ATLAS (2020) [75]	Excluded if HBsAg+ or if HBcAb+ HBsAb− HBVDNA+	Switch to long acting CAB + RPV vs. continue oral ART		4 (1%) in long-acting arm vs. 1 (<1%) in oral arm	1 acute hepatitis B in long-acting arm vs. 0 in oral arm
ATLAS-2M (2020) [76]	Excluded if HBsAg+ or HBcAb+ HBsAb− HBVDNA+	Long acting CAB + RPV every 8 weeks vs. every 4 weeks	522 (every 8 weeks), 523 (every 4 weeks)	2 (<1%) in 4-weeks arm vs. 5 (1%) in 8-weeks arm	2 hepatitis B in long-acting arm vs. 0 in oral arm
FLAIR (2020) [77]	Excluded if HBsAg+	Switch to long acting CAB+RPV vs. continue ABC/3TC/DTG	283 (long acting), 283 (oral ART)	7 (2%) in long acting arm vs. 2 (1%) in oral ART arm	2 acute hepatitis B (arm unspecified)
DUALIS (2020) [78]	Excluded if HBsAg+ (28% HBcAb+)	Switch to DTG+DRV/r vs. DRV/r +2 NRTI	131 (DTG arm), 132 (NRTI arm)	Not reported	None reported
PROBE 2 (2020) [79]	Excluded if HBsAg+	Switch to RPV+DRV/c vs. continue current ART	80 (RPV+DRV/c), 80 current ART	Not reported	0 in all arms
SOLAR (2023) [80]	Excluded if HBsAg+ or HBcAb+ HBsAb−	Switch to long acting CAB+RPV vs. continue TAF/FTC/BIC	447 (long acting), 223 (oral ART)	2 in CAB+RPV arm vs. 0 in TAF/FTC/BIC (only serious adverse events deemed to be drug-related were reported)	None reported
CARES (2024) [81]	Excluded if HBsAg+ or HBcAb+	Switch to long acting CAB+RPV vs. continue oral ART including TDF+ 3TC or FTC	255 (CAB+RPV), 257 (continue)	0 in both arms	0 in both arms
D2EFT (2024) [82]	Excluded if HBsAg+	Switch to DVR/r+DTG vs. DRV/r +2 NRTI vs. DTG +TDF+3TC/FTC from failing 1st line ART	271 (DRV/r+DTG), 262 (DRV/r +2 NRTI), 295 (DTG+TDF/XTC)	3 in DRV/r+DTG arm vs. 3 in DTG+TDF+ 3TC/FTC arm vs. 1 DRV/r + 2 NRTI arm	1 acute hepatitis B in DRV/r+DTG arm vs. 0 in other arms
ILLUMINATE Switch A NCT04223778 (2024) [83]	Excluded if HBsAg+ or HBV-DNA positive	Switch to ISL/DOR vs. continue current ART	672 (ISL/DOR), 336 (current ART)	4 (any grade) in both arms. 1 grade ≥3 event considered treatment related in current ART arm	1 acute hepatitis B in ISL/DOR vs. 0 in current ART arm (no recurrent HBV infection in both arms)
ILLUMINATE Switch B NCT04223791 (2024) [84]	Excluded if HBsAg+ or HBV−DNA+	Switch to ISL/DOR vs. continue TAF/FTC/BIC	322 (ISL/DOR) of whom 77 HBcAb+HBsAb+ and 10 HBcAb +HBsAb-, 321 (TAF/FTC/BIC)		0 acute hepatitis B in both arms 2 HBcAb+ HBsAb- patients experienced low level HBVDNA replication with normal liver function tests in ISL/DOR arm (1 discontinued, 1 spontaneous clearance). Both mounted HBsAb response
ARTISTRY-1 (2025) [85]	Excluded if HBsAg+ or HBcAb+ HBsAb−	Switch to oral LEN/BIC vs. continue current ART	103 (LEN/BIC), 25 current ART	None reported	0 in both arms

3TC, lamivudine; ART, antiretroviral treatment; BIC, bictegravir; CAB, cabotegravir; DOR, doravirine; DRV/c, cobicistat-boosted darunavir; DRV/r, ritonavir-boosted darunavir; DTG, dolutegravir; EFV, efavirenz; FTC, emtricitabine; HBcAb, hepatitis B core Antibody; HBsAb, hepatitis B surface antibody; HBsAg, hepatitis B surface antigen; HBV, hepatitis B virus; ISL, islatravir; LEN, lenacapavir; LPV/r, lopinavir/ritonavir; NRTI, nuceloside reverse transcriptase inhibitor; RAL, raltegravir; RPV, rilpivirine; TAF, tenofovir alafenamide; TDF, tenofovir disoproxil fumarate.

Overall, these randomized data support the notion that, in carefully selected and closely monitored populations, discontinuation of HBV-active ART is associated with a low incidence of clinically significant HBV reactivation. At the same time, the occurrence of occasional acute hepatitis B cases – sometimes in trials not specifically designed to capture HBV outcomes – highlights that the risk is not negligible and may be underestimated, particularly in the absence of systematic HBV DNA monitoring.

In conclusion, the available evidence from case reports, cohort studies, and randomized trials suggests that HBV reactivation after discontinuation of HBV-active antiretroviral therapy in PWH with pOBI (HBsAg-negative, anti-HBc-positive) is a real but overall infrequent event. When reactivation occurs, it is rarely random. Rather, it appears to cluster in specific clinical and virological contexts that point to incomplete immune control or unrecognized persistence of replication-competent virus. Across the different lines of evidence reviewed, a consistent set of factors emerges as being associated with increased risk. These include a history of HBsAg positivity despite current seronegativity, the absence of protective anti-HBs antibodies, advanced or poorly controlled HIV infection with low CD4^+^ T-cell counts, and situations of additional immune suppression. In some cases, prior exposure to lamivudine as the sole HBV-active agent may possibly have contributed to select resistant or immune-escape variants. Conversely, in individuals without these features – those with durable HIV suppression, preserved CD4^+^ cell counts, and evidence of effective immune control – data from both observational cohorts and randomized switch trials indicate that the absolute risk of clinically relevant HBV reactivation is low. In these settings, isolated or transient low-level HBV DNA detectability has occasionally been observed, but its clinical significance remains uncertain, particularly in the absence of HBsAg seroreversion or biochemical hepatitis.

### Role of hepatitis B virus vaccination in individuals with isolated anti-hepatitis B core antigen

Although HBV reactivation has been reported even in individuals who are anti-HBs positive, the inverse association between anti-HBs titers and reactivation risk raises the question of whether HBV vaccination may mitigate this risk. While no prospective or interventional studies have established a causal link – nor demonstrated that anti-HBs positivity *per se* prevents reactivation rather than reflecting more effective immune conditions – HBV vaccination remains a reasonable and guideline-endorsed strategy in this setting. As a matter of fact, international guidelines currently recommend HBV vaccination for individuals with isolated anti-HBc positivity [67]. This recommendation is largely informed by a pivotal study, which showed that a substantial proportion of PWH with isolated anti-HBc can mount a robust anti-HBs response following vaccination [68]. In that study, nearly half of participants developed protective anti-HBs titers after a single vaccine dose, while the vast majority of initial nonresponders achieved seroprotection after completing a full vaccination schedule. Thus, a first dose of HBV vaccination in individuals with isolated anti-HBc positivity can have an important diagnostic and stratification value. A strong anamnestic response to a single vaccine dose likely reflects resolved HBV infection with waning immunity, whereas failure to respond may suggest a higher risk of occult HBV infection and risk of reactivation, warranting completion of a full vaccination course. Emerging evidence indicates that newer adjuvanted vaccines may further improve seroconversion rates in this population [69].

Despite this solid immunological rationale, there is currently no evidence that vaccination alone is sufficient to prevent HBV reactivation after discontinuation of lamivudine/emtricitabine and tenofovir. Nevertheless, vaccination may be pragmatically incorporated into a two-step strategy when considering ART switches that remove HBV-active agents. In this framework, documenting a robust anti-HBs response – ideally before the switch – may provide additional reassurance when discontinuing HBV-active drugs, although careful virological and biochemical monitoring remains mandatory, and the overall strategy should be considered largely expert-opinion based.

In conclusion, occult HBV infection represents a biologically and clinically relevant condition in PWH, sustained by the long-term persistence of replication-competent cccDNA despite apparent virological resolution and long-term antiviral treatment. The available evidence indicates that HBV reactivation after discontinuation of HBV-active antiretrovirals in individuals with serologically resolved HBV is a genuine but overall infrequent event, whose occurrence is largely shaped by individual immunological status, historical serological patterns, and the presence of additional risk factors. In individuals with well controlled HIV infection, preserved CD4^+^ cell counts, and no markers suggestive of incomplete HBV clearance, the absolute risk of clinically meaningful reactivation appears low, as supported by cohort data and randomized switch trials. These findings argue against uniform restrictions on treatment simplification strategies and instead support a risk-based approach that integrates HBV history, serology, and vaccination status into ART decision-making, while acknowledging that the available evidence remains limited and that many management decisions in this area currently rely on expert interpretation of heterogeneous data. Close postswitch monitoring higher risk profiles, such as those with an isolated anti-HBc serological pattern, incomplete CD4^+^ T-cell recovery, or a recent anti-HBs seroconversion, especially when the latter occurred while receiving nucleos(t)ide analogue therapy. Current guidelines and position papers recommend careful follow-up in this setting but do not define the optimal monitoring strategy in terms of tests, frequency, or duration. In practice, monitoring should not rely on HBV DNA alone, as very low or transient viremia may occur without clinical significance; instead, interpretation should integrate the magnitude of HBV DNA increase with transaminase levels and HBsAg dynamics. Given that most reported reactivation events occur within the first 6 months after treatment switch, more intensive monitoring including HBV DNA and HBsAg during this early period, followed by routine liver enzyme surveillance thereafter, may represent a pragmatic approach. Well designed prospective studies or carefully followed retrospective cohorts with stored samples will be needed to define the most effective and cost-efficient monitoring strategies.

## Acknowledgements

GL participated in temporary advisory boards or received honoraria for speaker activities from INSMED, Pfizer S.r.l., and ViiV Healthcare; AS received speaker fees from AbbVie and Gilead; MP participated to own events or temporary advisory boards or received travel grants from AbbVie, Gilead, GSK, and Pfizer; PB participated in advisory boards for Gilead Sciences, MSD, and ViiV Healthcare. The authors declare that these relationship did not influence the content or conclusions of the present work.

### Conflicts of interest

There are no conflicts of interest.
